# The role of parkin in monkey

**DOI:** 10.1093/procel/pwae064

**Published:** 2024-11-13

**Authors:** Pingping Song, Dimitri Krainc

**Affiliations:** Ken and Ruth Davee Department of Neurology, Northwestern University Feinberg School of Medicine, Chicago, IL 60611, United States; Ken and Ruth Davee Department of Neurology, Northwestern University Feinberg School of Medicine, Chicago, IL 60611, United States

Parkinson’s disease (PD) affects 1%–2% of people over the age of 65 years and is characterized by the loss of dopamine-producing neurons in the substantia nigra (SN), as well as the buildup of phosphorylated α-synuclein (pS129-α-syn) in Lewy bodies (Aarsland et al., 2021; Fujiwara et al., 2002). While mutations in genes like PTEN-induced kinase 1 (PINK1) and Parkinson disease (autosomal recessive, juvenile) 2, (PARK2) are known to cause early-onset PD (Kitada et al., 1998), mouse models of these mutations have not successfully replicated the key pathological features of the disease, such as neurodegeneration and pS129-α-syn accumulation (Goldberg et al., 2003). This gap in animal models motivated the current study to develop a new nonhuman primate model. Han et al. focused on exploring the pathogenesis of PD, particularly through the role of the parkin gene (*PARK2*) (Han et al., 2024). They investigated how parkin deficiency contributes to the neurodegeneration of vulnerable dopaminergic neurons and α-synuclein accumulation, two major hallmarks of PD.

Using clustered regularly interspaced short palindromic repeats (CRISPR)/CRISPR-associated protein 9 (Cas9) technology targeting the parkin gene in fertilized eggs, they found that there was no significant loss of neuronal and mitochondrial proteins in the cortex, striatum, and SN of the parkin mutant (exon 2 and exon 3 deletion) monkey compared with the wild-type monkey at 3 years of age, suggesting that parkin mutations do not trigger neuronal loss in the developing monkey brain, which was different from PINK1 mutant monkey that exhibited neurodegeneration in developing and adult monkeys (Yang et al., 2019). It has been well established in prior *in vitro* studies that Parkin functions in the same pathway with PINK1, such as the PINK1-dependent mitophagy pathway (Narendra et al., 2008). However, the observed distinct phenotypes between PINK1 and parkin-deficient monkeys observed in this study suggest diverse functions of these two proteins. Moreover, the subcellular distribution of parkin and PINK1 in the primate brain under physiological conditions appears to be uncorrelated (Liu et al., 2025). These findings are consistent with other recent studies in human dopaminergic neurons that highlighted PINK1-independent functions of parkin, whereby parkin was activated by Ca^2+^/calmodulin-dependent protein kinase II (CAMKII) and dynamically recruited onto synaptic vesicles in PINK1 deficient neurons (Song et al., 2023).

Due to the extended period (4–5 years) of sexual maturity in monkeys, Han et al., (2024) did not investigate age-dependent pathological changes in monkeys with genetic editing at the fertilization stage. Alternatively, they used a stereotaxic injection of Adeno-associated virus (AAV) AAV9 to express parkin gRNA and Cas9 in the cortex and SN of adult monkeys at young ages (6–8 years) or old age (25–28 years). They observed a reduction of TH-positive neurons in the AAV parkin gRNA/Cas9–injected SN. However, targeting parkin in the cortex of 6- and 8-year-old monkeys via injection of AAV parkin Guide RNA (gRNA)/Cas9 did not induce significant loss of neuronal cells as compared with the control gRNA/Cas9 injection, suggesting the brain-region specific effects of parkin deficiency.

Han et al., (2024) conducted further biochemical characterization of monkey brain proteins with the 9-year-old monkey targeted by control gRNA or parkin gRNA in the substantia nigra. Interestingly, they found that parkin and its phosphorylated form pS65-parkin were reduced in the parkin-targeted SN, accompanied by the reduction of neuronal proteins tyrosine hydroxylase (TH) and Synaptosomal-associated protein 25 (SNAP25), suggesting that parkin deficiency can cause degeneration of substantia nigral dopaminergic neurons in adult monkeys. In contrast, they did not observe the alterations in mitochondrial proteins, such as mitofusin-2 (MFN2), optic atrophy 1 (OPA1), voltage-dependent anion-selective channel 1 (VDAC1), and dynamin-related protein 1 (Drp1), which were reported to be the substrates of parkin in immortalized cells (Gegg et al., 2010; Geisler et al., 2010; Wang et al., 2011), suggesting the distinct dominant function of parkin under physiological conditions in a primate model.

Han et al., (2024) demonstrated that phosphorylated parkin (pS65-parkin), mediated by PINK1, plays a key role in preventing the accumulation of phosphorylated α-synuclein (pS129-α-syn), a hallmark of PD pathology. When parkin is not phosphorylated, it leads to increased insolubility of parkin and accumulation of toxic α-synuclein. They found an age-related decline in parkin phosphorylation. Whether the decreased phosphorylation of parkin is due to decreased PINK1 level needs to be further studied. As the monkeys aged, parkin phosphorylation decreased, which coincided with an increase in pS129-α-syn levels. This suggests that aging exacerbates parkin-related neurodegenerative processes, implicating it as a key contributor to PD progression. Overexpression of wild-type parkin, but not a mutant form that cannot be phosphorylated by PINK1, effectively reduced α-synuclein accumulation. This highlights the potential of targeting parkin phosphorylation as a therapeutic approach in PD.

Importantly, Han et al., (2024) found that in the parkin-deficient monkeys, SN neurons were preferentially vulnerable which is consistent with the human pathology. Other recent studies show that PINK1 expression and its phosphorylation of parkin were detected only in monkeys, not in mice and pigs (Chen et al., 2024), further validating the importance of nonhuman primate models for studies of PD. By contrast, it has been well-established that modeling genetic forms of PD in mice has not reproduced the degeneration of nigral dopaminergic neurons that is observed in human disease. To resolve this discrepancy between rodent and human pathology, another study showed that only human but not rodent dopaminergic neurons exhibited an accumulation of toxic oxidized dopamine (Burbulla et al., 2017), suggesting that differences in dopamine metabolism in rodent brains contribute to their resistance to degeneration in genetic models of PD. These findings are consistent with the absence of neuromelanin in rodent nigra that is highly abundant in human and monkey brains. Together, these studies in part explain why preclinical studies in rodent models have not translated to human clinical trials.

In sum, Han et al., (2024) created *parkin*-deficient monkeys and observed age-dependent neurodegeneration and α-synuclein accumulation, particularly in the SN. This study represents a significant advance in modeling PD in nonhuman primates and opens new avenues for potential therapeutic strategies targeting parkin activation to reduce α-synuclein toxicity in PD. As part of future studies, it will be of great interest to further examine the mechanism of parkin-mediated effects on α -synuclein homeostasis.
